# Rapid purification of gram quantities of β-sitosterol from a commercial phytosterol mixture

**DOI:** 10.1186/1756-0500-7-182

**Published:** 2014-03-27

**Authors:** Narayanan Srividya, Deanna Brooke Heidorn, Bernd Markus Lange

**Affiliations:** 1 Institute of Biological Chemistry and M.J. Murdock Metabolomics Laboratory, Washington State University, Pullman, WA 99164, USA

**Keywords:** β-Sitosterol, Purification, Fractional crystallization, Chromatography, Phytosterol, Zeolite

## Abstract

**Background:**

β-Sitosterol, a plant sterol or phytosterol, has commercial uses in the nutraceutical and pharmaceutical industries, but is also employed frequently in biological research. Phytosterols always accumulate as mixtures, and obtaining highly pure β-sitosterol in larger quantities for biological assays has been a challenge.

**Findings:**

An improved method for the rapid purification of β-sitosterol from a commercial phytosterol extract is presented. Fractional crystallization of soybean oil yielded a soluble and an insoluble fraction. β-Sitosterol was purified by silica gel and Na-Y zeolite chromatography.

**Conclusion:**

The rapid and cost effective three-step purification described here afforded β-sitosterol in gram quantities with high purity (>92%) and yield (>22%).

## Findings

### Background

Plant sterols, also referred to as phytosterols, are important structural components of the plasma membrane and are involved in the regulation of various developmental processes, including those mediated by lipid hormones [1-3]. Phytosterols have been used successfully and safely for several decades to lower plasma cholesterol levels, and many margarines, butters, breakfast cereals and spreads are now enriched with plant-derived sterols and their esters [4,5]. While considerable progress has been made with obtaining specific phytosterols in pure form, the costs are still very high when larger amounts of pure sterols are required for structure-function studies in the nutritional, pharmaceutical and plant biology fields.

**Figure 1:**
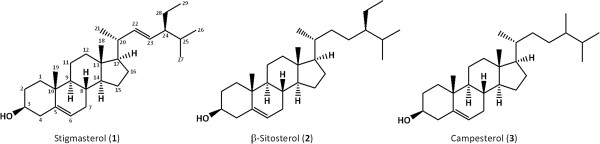
**Structures of phytosterols.** Common numbering of carbon atoms shown for stigmasterol (1).

Two general strategies for obtaining gram quantities of individual phytosterols have been pursued. Stigmasterol **1**, the most accessible and least expensive phytosterol, can be converted to β-sitosterol **2**, a phytosterol commonly used as a model for this class of plant metabolites, by selective hydrogenation or reduction of the Δ^22–23^ alkene, while protecting the Δ^5–6^ double bond (Figure 1) [6-8]. Even more cost-effective is the isolation of β-sitosterol from vegetable oils, but obtaining pure metabolites from mixtures containing structurally related phytosterols has been challenging. The simplest isolation approach is based on a series of crystallizations, but the purity of **2** is maximally in the 70% range [6,9-11]. Further purification to >90% purity can be achieved with chromatography over silica gel or Na-Y zeolite, but these protocols require repeated, time-consuming cycles of column purification [12-15]. Here we report a facile and rapid method that combines silica gel and Na-Y zeolite chromatography to afford **2** from a vegetable oil-derived phytosterol mixture at high yield (22.5%) and purity (94.2%).

### Materials

Crude soybean oil extract (termed “> 40% sitosterol”; S5753) was obtained from Sigma-Aldrich (St. Louis, MO). Solvents (acetone, chloroform, diethyl ether, ethanol, ethyl acetate, hexane, and toluene) were purchased from Fisher Scientific (Waltham, MA). Silica gel for column chromatography (type 60 Å; 100–200 mesh, 75–150 μm particle size) was obtained from Mallinckrodt (St. Louis, MO). Na-Y zeolite was bought from Sigma-Aldrich (St. Louis, MO) and activated using a Vulcan 3–130 bench top furnace at 500ºC for 18 h before use. The stirring speed for batch incubations of Na-Y zeolite with phytosterol mixtures was controlled using a PCT900 tachometer (General Tools, New York, NY) so that consistent but slow stirring occurred. Faster stirring speeds led to undesirably high concentrations of campesterol in solution. The Na-Y zeolite was reactivated by furnace heating as above.

### Results and discussion

**Figure 2:**
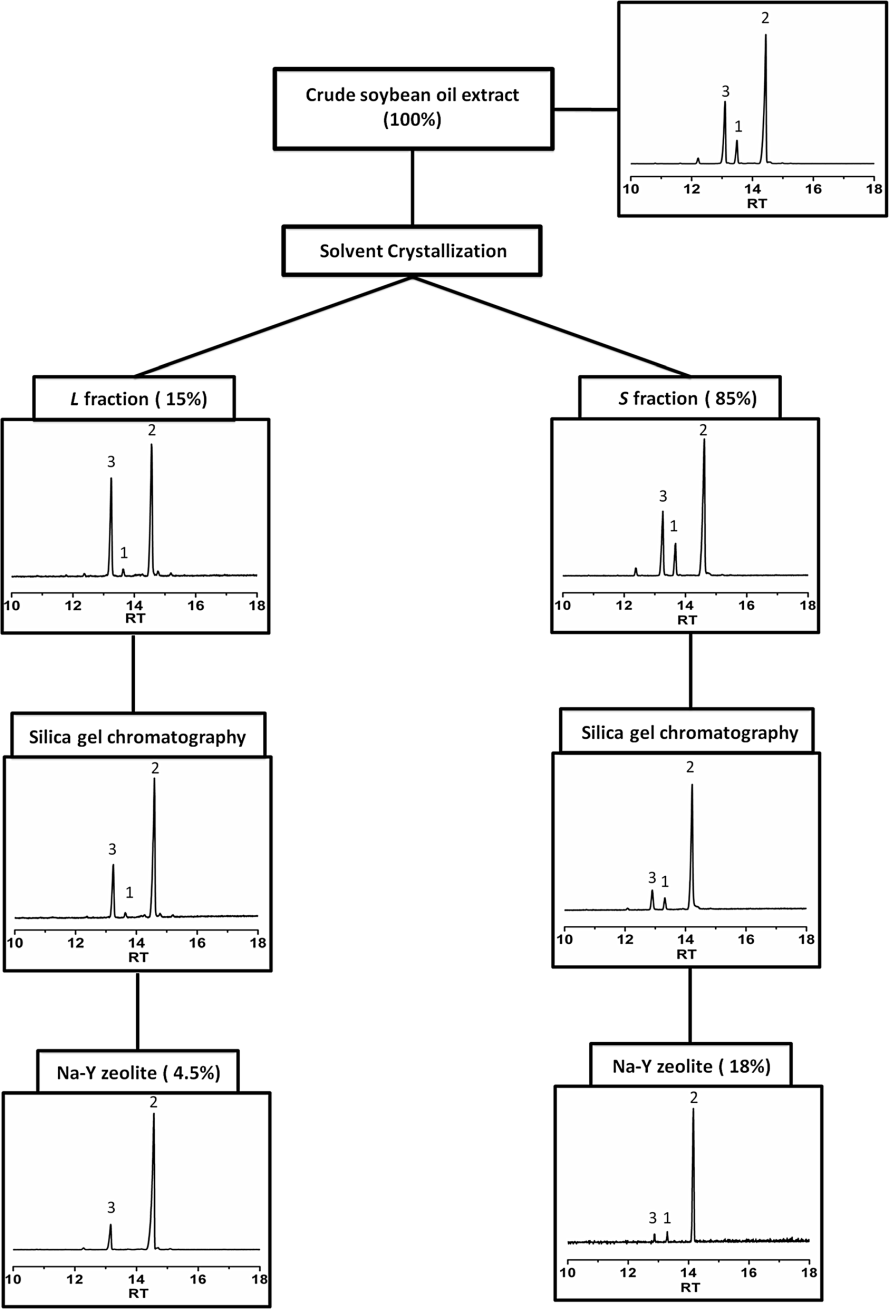
**Flow chart of β-sitosterol purification from a crude soybean oil extract.** Representative GC-MS chromatograms are shown for each step. Yields are provided in parentheses. Numbering of metabolites as in Figure 1.

Fractional crystallization has been used by various groups to obtain a solid (***S***) and a liquid (***L***) phytosterol fraction, of which only the ***L*** fraction (fairly low phytosterol content but with few other contaminants) was further processed. The phytosterol-rich ***S*** fraction contained high amounts of **1**, which was very difficult to remove when **2** was the primary target, and was thus discarded [6,9-11]. To avoid substantial losses of **2**, we first tested several solvents for obtaining an ***L*** fraction with a significant reduction in **1**, while maintaining options to also process the ***S*** fraction for purifying **2**. The solvent selection was based on previously published reports and included (1) acetone, (2) hexane/toluene/ethanol (4:2:1; v:v:v) and (3) diethyl ether (Table 1) [6,9-11]. Crude soybean oil extract was dissolved in 500 ml of each solvent and placed at −80°C overnight. After removal from the freezer, the contents were immediately filtered using a Büchner funnel with sintered glass insert to yield an ***L*** and an ***S*** fractions (Figure 2). The solvent of the ***L*** fraction was evaporated, and the ***S*** and dried ***L*** fractions separately dissolved in 10 ml chloroform. At this stage, an aliquot from each fraction was analyzed by GC-MS [16]. The use of diethyl ether resulted in the most significant reduction of **1** in the ***L*** fraction (Table 1). Campesterol (**3**) was enriched but can be more easily removed using column chromatography [12-14]. None of the solvents were effective in differentially reducing the amounts of **1** in the ***S*** fraction, while diethyl ether was the most appropriate solvent for maintaining high quantities of **2** (Table 1). All further experiments were thus performed using diethyl ether as a solvent. Phytosterols were distributed among the ***S*** and ***L*** fractions at 85% and 15%, respectively (Figure 2).

**Table 1:** Purification of β-sitosterol (1) from crude soybean oil extract

**Purification step**	**Fraction**	**Stigmasterol**	**β-Sitosterol**	**Campesterol**
		**(1)**	**(2)**	**(3)**
		**[% of total phytosterols]**		
Crude soybean oil extract (S5753, Sigma-Aldrich)				
	n.a.	9.3	60.7	27.8
Fractional crystallization				
Acetone	***S***	8.5	51.2	40.3
	***L***	12.3	57.9	29.8
Hexane/toluene/	***S***	12.3	57.7	30.1
ethanol (4:2:1)	***L***	4.5	66.0	29.5
Diethylether	***S***	12.4	61.5	26.2
	***L***	3.0	58.1	39.0
Silica column chromatography				
	***S***	7.1	78.2	14.7
	***L***	1.5	82.0	16.5
Na-Y zeolite chromatography				
	***S***	4.1	92.2	3.7
	***L***	0.1	94.2	5.7

Values represent averages of replicated experiments (n = 3–5).

The ***S*** and ***L*** fractions were then loaded separately onto silica gel columns, which were operated by gravity flow, and phytosterols eluted with hexane/ethyl acetate (6:1; v:v) as eluent (Figure 2). The flow rate was adjusted to 1 ml/min. Fractions (10 ml) were recovered and constituents analyzed by GC-MS [16]. The elution order was **2**, **1** and then **3**. Fractions 18–30, which contained ≥ 70% β-sitosterol, were pooled. The solvent of the combined β-sitosterol-enriched fractions was evaporated and the remainder dissolved in 200 ml hexane. The phytosterol content was again determined by GC-MS [16] and indicated a yield of phytosterols of 30%. The phytosterol mixture derived from the ***S*** fraction, after silica gel chromatography, contained a significantly increased proportion of **2** (78.2%), with decreased amounts of **1** and **3** (7.1% and 14.7%, respectively) (Table 1). The silica gel-purified phytosterol mixture from the processed ***L*** fraction contained even higher amounts of **2** (82.0%) and a particularly low amount of **1** (1.5%). Previous attempts to further purify **2** employed multiple (8 to 10) cycles of silica gel chromatography [14]. Since this protocol is very time-consuming, we evaluated if the purification could be sped up by employing only one more purification step but with a different stationary phase.

Prior work using Na-Y zeolite involved large amounts of material (60:1, w:w; adjusted based on total phytosterol amount) [14,15]. This method also required a high torque mixer and several manual sample loadings (multiple cycles). We lowered the activated Na-Y zeolite amount to 10 : 1 (w : w) and performed only a one-step purification with phytosterol extracts retrieved after silica gel chromatography (***S*** and ***L*** fractions processed separately). The mixture was stirred at 200 rpm at 32°C for 48 h (Figure 2). The zeolite was filtered off and the solvent of the filtrate removed *in vacuo*. The sterol composition was assessed by GC-MS [16]. The target metabolite **2** was obtained with high purity from both the ***L*** (94.2%) and ***S*** (92.2%) fractions (Table 1). The overall yield of **2**, when both the ***S*** and ***L*** fractions were processed, was 22.5%, which is a significant increase over the *status quo*, where the ***L*** fraction yields roughly 13% of the target metabolite [12-15]. The selectivity of Na-Y zeolite for removing **3** is quite remarkable. In this microporous chromatographic material, SiO_4_ and Al_2_O_3_ tetrahedrae form pores with a uniform diameter of 7.4 Å. Molecular modeling studies by Berezin and colleagues [12] indicated that **3** has a diameter of 6.3 Å, which allows its entry into the pores. In contrast, **1** and **2** have slightly larger diameters of 7.7 and 7.5 Å, respectively, which is too large for uptake, and these compounds therefore remain in solution.

In conclusion, we have demonstrated that, using a combination of fractional crystallization, silica gel and Na-Y zeolite chromatography, the purification of **2** at high purity from a crude vegetable oil extract can be completed in 72 h with high overall yield. Considering the demand of **2** for clinical trials and biological structure-function studies, this protocol allows for the rapid production of the highly pure metabolite at an affordable price.

## Abbreviations

S: Solid fraction; L: Liquid fraction; GC-MS: Gas chromatography–mass spectrometry.

## Competing interests

The authors declare that they have no competing interests.

## Authors’ contributions

NS and BML designed the experiments and wrote the manuscript. NS and DH carried out the experiments. All authors read and approved the final manuscript.
